# Supplementary material for: Effects of Short-Term Low Energy Availability on Metabolism and Performance-Related Parameters in Physically Active Adults
Source: Nutrients. 2025 Jan 14;17(2):278. doi: 10.3390/nu17020278 (PMC11767613; doi:10.3390/nu17020278)
Supplement: Supplementary file 1 [file nutrients-17-00278-s001.zip › Nolte_Supplementary Material S1.pdf]

Supplementary Material S1

Table S1. Descriptive data of energy intake, energy expenditure and energy availability at baseline and during intervention.

			**EI**		**TEE**		**EI – TEE**		**EEE**		**EA**	
		Group	Kcal/day	Kcal/kg FFM/day	Kcal/day	Kcal/kg FFM/day	Kcal/day	Kcal/kg FFM/day	Kcal/day	Kcal/kg FFM/day	Kcal/day	Kcal/kg FFM/day
Baseline	Day 1	45	2307 ± 780	42.06 ± 7.24	2898 ± 730	53.31 ± 5.92	-592 ± 625	-11.25 ± 11.58	476 ± 406	7.91 ± 5.61	1831 ± 504	34.15 ± 7.90
		30	2749 ± 694	41.23 ± 6.87	3347 ± 598	50.45 ± 5.97	-598 ± 548	-9.22 ± 8.78	432 ± 455	6.41 ± 6.95	2317 ± 794	34.83 ± 10.44
		10	2420 ± 827	40.46 ± 8.58	2803 ± 353	48.17 ± 5.61	-382 ± 604	-7.72 ± 10.80	117 ± 223	1.92 ± 3.56	2304 ± 745	38.54 ± 6.81
	Day 2	45	2293 ± 698	42.09 ± 6.65	3235 ± 514	60.57 ± 8.03	-942 ± 708	-18.48 ± 13.78	803 ± 383	14.92 ± 6.90	1489 ± 829	27.17 ± 12.66
		30	2702 ± 535	40.66 ± 5.83	3396 ± 538	51.67 ± 8.30	-694 ± 612	-11.01 ± 10.51	461 ± 454	7.30 ± 7.37	2241 ± 704	33.36 ± 8.70
		10	2441 ± 907	40.35 ± 6.22	3203 ± 611	54.71 ± 7.07	-761 ± 478	-14.36 ± 9.92	404 ± 415	6.25 ± 4.86	2038 ± 675	34.10 ± 7.95
	Day 3	45	2310 ± 657	42.59 ± 6.57	3151 ± 560	58.56 ± 5.58	-841 ± 607	-15.98 ± 11.21	610 ± 421	11.00 ± 7.03	1700 ± 704	31.60 ± 11.74
		30	2774 ± 832	40.89 ± 6.60	3211 ± 451	48.78 ± 6.80	-437 ± 678	-7.89 ± 11.98	183 ± 295	2.67 ± 4.19	2592 ± 845	38.22 ± 8.25
		10	2245 ± 117	37.26 ± 5.42	3520 ± 951	59.40 ± 9.64	-1274 ± 340	-22.13 ± 6.74	596 ± 809	10.34 ± 12.76	1649 ± 1053	26.93 ± 11.34
	Means	45	2303 ± 702	42.25 ± 6.29	3095 ± 579	57.48 ± 5.34	-792 ± 601	-15.23 ± 11.12	630 ± 329	11.28 ± 4.77	1674 ± 654	30.97 ± 10.00
		30	2742 ±616	40.93 ± 4.09	3318 ± 464	50.30 ± 5.85	-576 ± 450	-9.37 ± 8.10	358 ± 325	5.46 ± 5.08	2383 ± 688	35.47 ± 6.95
		10	2369 ± 786	39.36 ± 4.50	3175 ± 612	54.09 ± 6.25	-806 ± 349	-14.74 ± 7.64	372 ± 325	6.17 ± 4.73	1997 ± 690	33.19 ± 3.94
Intervention	Day 1	45	2735 ± 628	50.37 ± 2.14	2772 ± 535	61.60 ± 7.11	-37 ± 5	-1.23 ± 6.41	307 ± 118	5.59 ± 1.20	2427 ± 519	44.78 ± 1.20
		30	2426 ± 458	36.45 ± 3.88	3257 ± 388	49.37 ± 4.79	-830 ± 213	-12.95 ± 4.80	431 ± 149	6.36 ± 1.26	1995 ± 341	30.10 ± 3.54
		10	959 ± 149	16.42 ± 1.89	3151 ± 653	53.52 ± 5.09	-2192 ± 545	-37.10 ± 5.05	402 ± 92	6.93 ± 1.73	557 ± 95	9.49 ± 0.67
	Day 2	45	2692 ± 799	49.01 ± 4.74	2618 ± 732	48.23 ± 8.47	73 ± 476	0.78 ± 8.76	363 ± 220	6.36 ± 2.36	2329 ± 596	42.65 ± 3.18
		30	2364 ± 484	35.37 ± 2.41	3310 ± 671	49.77 ± 6.06	-946 ± 296	-14.40 ± 4.61	520 ± 284	7.57 ± 3.17	1843 ± 312	27.80 ± 2.95
		10	963 ± 168	16.47 ± 2.13	3029 ± 559	51.56 ± 3.86	-2066 ± 452	-35.09 ± 4.06	422 ± 87	7.25 ± 1.53	541 ± 100	9.22 ± 0.90
	Day 3	45	2708 ± 669	49.75 ± 2.28	2939 ± 448	55.03 ± 7.14	-231 ± 431	-5.29 ± 7.88	340 ± 151	6.10 ± 1.39	2368 ± 529	43.65 ± 1.76
		30	2288 ± 313	34.60 ± 4.79	3360 ± 471	50.99 ± 6.26	-1072 ± 431	-16.39 ± 5.57	405 ± 100	6.03 ± 0.74	1884 ± 238	28.57 ± 2.86
		10	946 ± 151	16.15 ± 1.22	3013 ± 482	51.66 ± 7.19	-2066 ± 396	-35.51 ± 6.96	394 ± 79	6.71 ± 0.88	552 ± 87	9.44 ± 0.83
	Day 4	45	2675 ± 703	48.99 ± 2.97	2802 ± 436	52.25 ± 4.56	-127 ± 344	-3-26 ± 5.86	341 ± 152	6.11 ± 1.40	2334 ± 560	42.88 ± 4.21
		30	2298 ± 323	34.74 ± 2.93	3347 ± 358	50.99 ± 6.51	-1049 ± 296	-16.25 ± 5.79	425 ± 107	6.35 ± 1.01	1873 ± 251	28.39 ± 2.86
		10	954 ± 184	16.22 ± 1.28	3160 ± 648	53.84 ± 6.59	-2206 ± 514	-37.63 ± 6.55	397 ± 103	6.73 ± 1.01	557 ± 95	9.49 ± 0.79
	Day 5	45	2710 ± 601	49.93 ± 2.79	2813 ± 527	52.14 ± 4.39	-104 ± 221	-2.22 ± 3.81	321 ± 74	5.93 ± 0.84	2389 ± 541	44.00 ± 2.49
		30	2251 ± 410	33.90 ± 3.31	3508 ± 354	53.43 ± 6.53	-1257 ± 221	-19.53 ± 5.49	398 ± 76	6.02 ± 0.91	1853 ± 368	27.88 ± 3.25
		10	998 ± 245	16.85 ± 1.67	3226 ± 371	55.76 ± 8.63	-2228 ± 296	-38.92 ± 8.91	397 ± 103	6.73 ± 1.01	601 ± 368	10.12 ± 0.84
	Means	45	2704 ± 675	49.6 ± 2.4	2789 ± 503	51.9 ± 5.1	-85 ± 329	-2.2 ± 5.6	335 ± 137	6.0 ± 1.2	2369 ± 545	43.6 ± 1.7
		30	2326 ± 358	35.0 ± 1.8	3356 ± 405	50.9 ± 5.0	-1031 ± 184	-15.9 ± 4.2	436 ± 129	6.5 ± 1.1	1890 ± 262	28.5 ± 2.1
		10	964 ± 173	16.4 ± 1.4	3116 ± 504	53.3 ± 5.2	-2152 ± 376	-36.8 ± 5.0	403 ± 81	6.9 ± 1.0	561 ± 101	9.6 ± 0.5

Values are presented in means ± SD. EI = energy intake; TEE = recorded 24-h total energy expenditure; EEE = exercise energy expenditure; EA = energy availability.
