# Supplementary material for: Effects of Short-Term Low Energy Availability on Metabolism and Performance-Related Parameters in Physically Active Adults
Source: Nutrients. 2025 Jan 14;17(2):278. doi: 10.3390/nu17020278 (PMC11767613; doi:10.3390/nu17020278)
Supplement: Supplementary file 1 [file nutrients-17-00278-s001.zip › Nolte_Supplementary Material S2.pdf]

Supplementary Material S2

Table S2. Resting metabolic rate before and after treatments.

	Pre	Post	Delta	P-value
RMR (kcal/day)				0.117
45	1926 ± 355	1910 ± 337	-16 ± 234	
30	2155 ± 266	2083 ± 282	-72 ± 188	
10	2094 ± 344	1777 ± 364	-316 ± 396	
RMR (kcal/kg FFM/day)				0.310
45	35.74 ± 3.23	35.58 ± 4.18	-0.16 ± 4.63	
30	34.57 ± 4.73	33.38 ± 2.94	-1.19 ± 2.95	
10	35.73 ± 3.06	31.19 ± 6.51	-4.54 ± 6.95	

Values are presented in means ± SD. RMR = Resting metabolic rate. P-value represent the results of ANOVA.
