# Supplementary material for: Effects of Short-Term Low Energy Availability on Metabolism and Performance-Related Parameters in Physically Active Adults
Source: Nutrients. 2025 Jan 14;17(2):278. doi: 10.3390/nu17020278 (PMC11767613; doi:10.3390/nu17020278)
Supplement: Supplementary file 1 [file nutrients-17-00278-s001.zip › Nolte_Supplementary Material S3.pdf]

Supplementary Material S3

Table S3. Energy expenditure and required energy availability of each participant over 8 days.

	**24-h EE**	**EEE**	**rEA**	**rEA**
	**Kcal**	**Kcal**	**Kcal**	**Kcal/kg FFM/day**
1	3329 ± 233	317 ± 220	3012 ± 185	56,19
2	3156 ± 447	622 ± 646	2533 ± 229	39,95
3	4232 ± 493	495 ± 372	3737 ± 585	41,94
4	2725 ± 159	348 ± 162	2377 ± 111	42,75
5	2995 ± 421	341 ± 157	2654 ± 367	51,14
6	2704 ± 123	306 ± 129	2398 ± 112	48,65
7	2824 ± 184	309 ± 181	2514 ± 150	48,17
8	3108 ± 179	319 ± 269	2789 ± 136	43,24
9	3169 ± 184	339 ± 138	2831 ± 146	43,95
10	2778 ± 267	234 ± 144	2544 ± 206	52,46
11	2969 ± 338	296 ± 219	2673 ± 256	41,50
12	3978 ± 293	475 ± 180	3503 ± 268	40,60
13	3157 ± 175	411 ± 166	2746 ± 156	51,42
14	3660 ± 205	270 ± 168	3390 ± 185	48,50
15	3825 ± 345	595 ± 273	3230 ± 153	38,41
16	3431 ± 371	722 ± 414	2709 ± 166	41,30
17	3625 ± 233	755 ± 244	2870 ± 97	38,78
18	2468 ± 249	394 ± 315	2074 ± 138	41,40
19	2219 ± 287	243 ± 138	1977 ± 212	49,05
20	3145 ± 364	600 ± 363	2545 ± 162	46,62
21	3057 ± 224	313 ± 52	2745 ± 215	52,19
22	2906 ± 448	368 ± 204	2538 ± 407	47,80
Mean value	3157	412	2745	45.73
Std. deviation	488	154	428	5.10
Minimum	2219	234	1977	38.41
Maximum	4232	755	3737	56.19

Values are presented in means ± SD. 24-h EE = recorded 24-h total energy expenditure; EEE = exercise energy expenditure; rEA = required energy availability to cover energy expenditure of daily living without exercise.
